# State of evidence on municipal strategies for health promotion and prevention: a literature and database research (Scoping Review)

**DOI:** 10.1186/s12889-022-12607-0

**Published:** 2022-02-14

**Authors:** Eike Quilling, Vivien Mielenbrink, Anke Osterhoff, Stefanie Terhorst, Patricia Tollmann, Stefanie Kruse

**Affiliations:** 1 Department of Applied Health Sciences, University of Applied Sciences, Gesundheitscampus 6-8, 44801 Bochum, Germany; 2 Department of Social Work, University of Applied Sciences RheinMain, Kurt-Schumacher-Ring 18, 65197 Wiesbaden, Germany

**Keywords:** Health promotion, Healthy municipalities, Determinants of health, Implementation, Health equity, Health in all policies, Community, Sustainable overall strategies

## Abstract

**Background:**

There are gaps in the research regarding the implementation and evidence of overall strategies for municipal health promotion addressing communities. The aim of this scoping review is to gain initial findings concerning theoretical models, approaches and evidence on strategies of municipal health promotion, which include self-care, mutual aid and healthy environments. The findings can enrich the development of health promotion services.

**Methods:**

A systematic scoping literature analysis was conducted in the databases PubMed, Web of Science, SAGE-Journals, Wiley-Online, ScienceDirect, LIVIVO and WiSo database as well as in a German project database. Evaluation studies and research reports on strategies in municipal health promotion were included and analysed qualitatively.

**Results:**

According to our predefined inclusion and exclusion criteria, 15 hits were included. Capacity building, planning and the establishment of structures for health promotion were identified as theory-based models and approaches. None of the publications included showed clear evidence of the effects of municipal health promotion measures in terms of classically medically defined evidence.

**Conclusions:**

The use of evidence-based theoretical models and approaches is no guarantee for the success of strategies for municipal health promotion. Challenges with regard to evidence are the execution of study designs corresponding to higher evidence classes and the isolation of effects of health promotion measures in complex environments.

**Trial registration:**

This scoping review was not registered beforehand.

**Supplementary Information:**

The online version contains supplementary material available at 10.1186/s12889-022-12607-0.

## Background

From a public health and health policy perspective, social inequality plays a key role for the health and life expectancy of the population. People with low incomes, occupational status and educational levels are at higher risk for the development of chronic diseases or functional limitations in everyday life and quality of life [1–3]. Health promotion aims to contribute to health equity and includes the ability “to identify and to realize aspirations, to satisfy needs, and to change or cope with the environment”(4). An appeal for health promotion in living environments was first documented in the Ottawa Charter after the first international conference on health promotion of the World Health Organisation (WHO) [4]. According to this, health promotion is defined as "the process of enabling people to increase control over, and to improve, their health”. Epp [5] names self-care (or individual health decisions and actions), mutual aid (or supporting others to cope) and healthy environments (or creating conditions and environments that are beneficial to health) as three mechanisms that are inherent in health promotion. From there on, health promotion was increasingly considered by municipalities. As defined by Green and Kreuter, it is "any combination of educational and environmental supports for actions and conditions of living conducive to health" [6]. The purpose of health promotion is to enable people to gain greater control over the so-called determinants of health.

These determinants are factors that influence health in a threatening, promoting, or protective manner [7]. They can be categorized into overarching, interrelating areas, revealing different levels, that can potentially be affected by health policy-making [7]. These areas can be imagined as a series of layers on top of each other. The general socio-economic, cultural, and environmental conditions (i.e. social inequalities) form the outer layer. As macro-factors they represent the most complex determinants of health and are, in this sense, considered to be the "causes of the causes" of illness and impaired health. The next layer is formed by living and working conditions, which can include stress at work, education, housing situation and the health system. It is particularly noteworthy that these conditions (i.e. different employment opportunities or economic resources) have a higher explanatory power for health than health behaviour alone and that they can have both, an independent and mediating effect on health. The next layer includes individual integration in various social networks. Interpersonal support from friends, family, community health workers, healthcare workers, informal leaders or teachers encourages people to maintain and restore their health and mitigates the risk of external influences that are harmful to health. These determinants have a direct effect on health but also act to influence relevant behaviours indirectly. Lifestyle and health behaviour on the subsequent layer primarily refer to individual health-promoting as well as health-damaging behaviours (i.e. nutrition, tobacco and alcohol consumption). Predictors to these factors are internal determinants (i.e. knowledge, perception, self-efficacy, self-esteem). Finally, genetic dispositions, gender and age on the innermost layer also play a role in this model. In contrast to the other factors that can potentially be modified with prevention and health promotion strategies, they however represent unalterable, fixed determinants of health [7, 8].The consideration of the determinants of health constitutes an important approach to health promotion [7].

Neither the individual nor the health care sector should be held solely responsible for people’s health. Health should rather be a guiding principle of all political and social actions [9]. The Health in all Policies (HiaP) approach describes the need for living environment-oriented health promotion involving all policy areas and intersectoral networks [10]. The implementation of these approaches at a local level is of particular importance. The municipality is understood as the smallest administrative unit and a comprehensive system in which settings such as day care centres, schools, businesses, supply and leisure facilities are organized [11, 12]. Municipalities thus play a key role in sustainably implementing and anchoring local as well as cross-sectoral strategies for the promotion of health as well as health equity [13, 14]. The term ‘municipal health promotion’ describes coordinated activities with a general, overarching goal, e.g. promotion of physical activity. These activities are carried out in a variety of different settings with the involvement of various stakeholders (e.g. institutions, organisations, associations and companies within the private and public sector, political systems, academia, civil society and the media) [15]. Consequently, municipal health promotion can, for example, refer address the needs of the elderly in a quarter via the implementation of health-promoting cities [16]. To promote physical activity, a municipality could firstly raise awareness of the issue and provide information (e.g. in schools, day care centres, businesses). It could further consist of support and funding for clubs (especially sports clubs) to ensure low-threshold access to physical activity opportunities. With respect to urban development measures, it should create opportunities, spaces and infrastructural conditions that encourage physical activity (e.g. green spaces or sports areas such as swimming pools, cycle paths and footpaths) [17]. The German Prevention Act clearly emphasizes the importance of the municipality for health promotion and aims to further expand municipal responsibility for the promotion of living conditions conducive to health [18]. However, the latest prevention report clarifies that, despite positive examples of municipal health promotion and prevention programmes, there are still considerable gaps regarding the comprehensive anchoring of integrated municipal overall concepts and the evidence of these approaches [19]. Academia plays a crucial role within the public health system as it not only educates professionals that work in this sector but also conducts basic and applied research that is needed for policy development and evaluation [20]. Whilst there already is a considerable amount of available literature evaluating the effectiveness and cost-effectiveness of health promotion interventions addressing chronic diseases and especially non-communicable diseases and associated risk factors [21], little is known about theoretical models and approaches of how municipal health promotion interventions can be conceptualized, implemented and evaluated [22]. Babitsch et al. [23] come to a similar conclusion, noting a need to improve, for example, the use of planning procedures. Böhm [24] examined the goals, elements, potentials and barriers of preventive and health promotion policies at the municipal level. They found that despite the theoretical relevance of the municipality, there is a lack of information regarding the practical design and conditions for success of municipal health promotion and prevention policies. They derived five core elements of a targeted and coordinated municipal health promotion and prevention policy, namely detailed health reporting systems, municipal health and prevention concepts, integration of individual local government units, coordination of all actors in the policy field and, as a cross-sectional task, the participation of those affected. Thus, the authors stated, that it is still unknown whether these core elements are implemented and how municipalities can counter the described obstacles [24]. Merzel and D’Afflitti [25] assume that the development of integrated theories of community health change could be valuable for health promotion as a research field, as it could guide the development of multilevel programs. Besides this, they describe the need for valid evaluation methods that reflect the complexity of the process of community change.

Since the currently available literature is scarce, we approached the current state of knowledge by methodological means of a scoping review. Scoping reviews are particularly suitable when the research aims to gain an initial impression of the current literature, for example to derive temporary working definitions or to map the key concepts of a research area and prepare a future systematic review. In contrast to classical systematic reviews, scoping reviews provide an overview over the current literature independent of the respective quality. The value of scoping reviews for evidence-based practice lies in the broad examination of complex issues, clarification of key concepts and identification of insights about the types of evidence that are relevant for practice.

The aim of this literature and database review, conducted by Quilling and Kruse [26], therefore is to give a first overview of theoretical models and approaches of municipal health promotion and prevention by means of a scoping review. Due to the special importance of evidence-based approaches as a fundamental requirement for the planning of strategies, their consideration in the conception of overall strategies is of particular interest here. The aim is to clarify whether, how and to what extent evidence is taken into account, how authors understand the concept of evidence, which values are underlying and how they approach evidence. Consequently, two central research questions can be derived:

1. Which theoretical models and approaches are used in the conception of overall strategies of municipal health promotion?
2. To what extent is the concept of ‘evidence’ suited for overall strategies of municipal health promotion and what evidence of the effectiveness and implementation of these strategies is available?

The present article may serve as a basis for the development of measures for municipal health promotion and can further accompany the development of health promotion services. In addition, it encourages paying attention especially to strategies that are grounded on evidence-based theoretical models and approaches.

## Methods

To answer the research questions, between 15 June and 15 September 2018, we conducted a scoping review on evaluation studies and research reports on strategies in municipal health promotion (published between 2010 and 2018). For this purpose, the databases and portals PubMed, Web of Science, SAGE-Journals, Wiley-Online, ScienceDirect, LIVIVO and WiSo database as well as a German-language project database (Kooperation für nachhaltige Prävention und Präventionsforschung; prevention research cooperative) were searched.^1^ In order to develop a search strategy, basic literature was screened as a first step. Next, a search protocol, including the eligibility criteria (see Table 1), was defined. Subsequently, synonyms were tested and further terms were determined during the process of defining a search strategy. The final English and German language search terms were used in alternating combinations leading to as many matching hits as possible. For example, the search strategy was conducted in PubMed as follows:

**Table 1:** Inclusion and exclusion criteria for the Scoping Review

Inclusion and exclusion criteria for the Scoping Review
Only German and English language publications were included. Publications in other languages were excluded
Only publications published between 2010 and 2018 were included
Only reviews (narrative, systematic reviews and scoping reviews), empirical studies, evaluation and practice reports were included
Only publications reporting on municipal health promotion and prevention practices in developed countries were included
Only strategies and measures that promote health in municipalities were included
Only municipal overall strategies, but also district and neighbourhood-related measures were considered
All target groups were included
Primary prevention intervention designs were included
Publications with groups affected by social inequality were included
Publications that enable a valid appraisal of the effectiveness of the described strategies were included. Theoretical papers and recommendations for action were excluded

((((kommunal*[Title] OR kommune[Title] OR sozialraum*[Title] OR quartier*[Title] OR stadtteil*[Title] OR municipal*[Title] OR „community based “[Title] OR community-based[Title])) AND (Gesundheits*[Title] OR Gesundheitsförder*[Title] OR prävent*[Title] OR Prävention[Title] OR health promot*[Title] OR prevent*[Title])) AND (integrierte*[Title] OR Netzwerk*[Title] OR netzwerkorient*[Title] OR integrat*[Title] OR network*[Title] OR network-based[Title])) AND (Evidenz*[Abstract] OR Effekt*[Abstract] OR Evaluation*[Abstract] OR evaluier*[Abstract] OR Qualität*[Abstract] OR evidence[Abstract] OR effect[Abstract] OR „best practice “[Abstract] OR “good practice” [Abstract] OR evaluat*[Abstract] OR quality[Abstract] OR outcome[Abstract]).

A supplementary unsystematic online search, using the search terms above in various combinations, complemented the search strategy. An extension to further databases and grey literature did not occur. In the next step of the scoping review and following the search protocol, two independent reviewers carried out a screening of the results by applying the eligibility criteria and systematically analysed the abstracts regarding their suitability (see Additional file 1 Figure 1 for PRISMA diagram, Additional file 1). Theoretical models and approaches, the concept of evidence, the evidence classes as well as the evaluation tools applied in the included projects were mapped out in the qualitative content analysis.

The findings are presented in accordance with the Preferred Reporting Items for Systematic reviews and Meta-Analyses (PRISMA) statement [27] and the extension for scoping reviews [28]. No review protocol was registered beforehand.

## Results

The literature search yielded 978 initial hits. After removing duplicates and scanning titles, the pre-selection was reduced to 264 publications. In accordance with the PRISMA recommendations, the search protocol and the preliminary hit list were screened by both reviewers and checked for possible inconsistencies in the search strategies. The identified abstracts were then systematically analysed for their suitability for further inclusion in the evaluation process. After the application of the defined inclusion and exclusion criteria (see Table 1) 38 publications were analysed in full text. Fifteen of these evaluation studies and research reports were selected for further analysis. The characteristics of the included papers vary greatly. While some address a specific field of health promotion in the context of municipalities, others have a more general, wider view on prevention and health promotion and some focus more on the framework for municipal health promotion. In some included studies, the focus lies particularly on the promotion of health equity via, for example, low-threshold services [29–31]. Additional file 1 Figure 1 shows the results of the research and steps of inclusion based on the PRISMA scheme (see Figure 1, additional file 1).

Additional file 1 Figure 1 Flow chart of the research and selection process.^2^

After a brief introduction of the included publications, the results of the qualitative analysis will be systematically presented below. The findings concerning the available evidence on the effectiveness and implementation of the analysed strategies of municipal health promotion will be presented as they relate to the research questions.

### Theoretical models and approaches of municipal health promotion

The models and approaches that are categorised and introduced in this section are: capacity building, planning and the establishment of structures for health promotion. They were identified on the basis of the included publications and will be explained in more detail below. Table 2 presents an overview of the included publications and their underlying theoretical models and approaches.

**Table 2:** Overview of the included publications and theoretical models and approaches they contain

Publications	Theoretical models and approaches		
	Capacity building	Planning	Establishment of structures
Alisch and Freytag-Leyer [29]: Community Health Management to Enhance Behaviour (quarter development); Germany	✓		✓
Berg, Stolzenberg [30]: City district mothers; Germany	✓		✓
Böhme, Bunge and Preuß [32]: Urban environmental justice; Germany	✓	✓	
Frantz and Heinrichs [33]: Family-based prevention programmes; Germany	✓		
Große, Menkouo and Grande [34]: Sustainable strategies for district-related health promotion; Germany	✓	✓	✓
Hargreaves et al. [35]: Healthy Weight Collaborative; USA	✓	✓	
Harris and Sandor [36]: Strategy development of local health promotion (Delphi Study); Australia	✓		
Kegler, Rigler and Honeycutt [37]: Importance of planning in the context of municipal health promotion; USA		✓	
Larsen, Pedersen, Davies and Gulis [38]: Content planning of measures for municipal health promotion; Denmark		✓	
Magnus, Knudtsen, Wist, Weiss and Lillefjel et al. [39]: Methodical approach for planning municipal health promotion; Norway		✓	✓
Reimann, Böhme and Bär [40]: Health in the district (health promotion and urban development); Germany	✓	✓	✓
Rütten, Wolff and Streber [41]: Interactive Knowledge Transfer in Health Promotion; Germany	✓	✓	✓
Steenbakkers, Jansen, Maarse and de Vries [42]: Planning processes in the context of Health in all Policies; Netherlands	✓	✓	
Storm, Harting, Stronks and Schuit [43]: (Measuring-)stations of health in all policies at local (municipal) level; Netherlands		✓	
Trojan, Süß, Lorentz, Wolf and Nickel [44]: Quarter-related health promotion—capacity development in the community; Germany	✓	✓	✓

In the literature on health promotion, the term ‘capacity building’ is a collective term with many divergent definitions [45]. In this paper, the approach of capacity building is associated with strategies and concepts for developing the competencies of actors, intersectoral networking and enabling participation. Many of the strategies addressed in the included publications name capacity building as an objective. Intersectoral networking in particular plays a major role [32, 34, 40, 42, 44]. It is seen as a significant success factor for more environmental justice in the city [32] or as a basis for neighbourhood-related health promotion. To meet the challenges and obstacles of intersectoral networking, further training and coaching [42] or local round tables are proposed [44]. Some of the included studies emphasise the importance of knowledge management for capacity building. They also use evidence-based materials and training courses [35, 42]. For example, the provision of models, checklists and internet platforms for participating municipalities promotes a regular exchange between the actors [35]. In addition to the municipal actors, local residents can also receive information materials, since capacity-building effects are also attributed to this approach [40]. Another option is competence-building courses based on evidence-based explanation and action models [33]. Rütten et al. [41] propose a promising approach for projects in the shape of interactive knowledge transfer through intersectoral networking, knowledge exchange and participation by means of collaborative concept development. All included publications and practice projects mention the importance of participation either as a bottom-up strategy for planning measures with institutional partners [29, 32, 39–41, 44] or as an instrument for target-group and needs-oriented planning or adaptation of measures from the perspective of the users themselves [29, 30, 34]. The results show that participation processes fulfil three functions: First, identification with the developed measures by all actors, including the users; Second, needs-based planning from the users’ point of view, and thirdly enabling flexible adaptation of measures.

The concept of planning here includes the strategic approach to the development of concrete interventions in the community as well as research findings on special models and instruments for effective and easy planning processes in the context of health promotion. Thirteen of the fifteen publications included enhance the importance of planning in municipal health promotion measures but differ in their emphases and approaches. Frantz and Heinrichs [33], Hargreaves et al. [35] and Rütten et al. [41] developed their health promotion measures on the basis of topic-specific evidence-based models that were identified by means of a scientific literature review. Harris and Sandor [36] also mention evidence-based planning and decision-making as a key factor for sustainable health-related changes. All included publications use participatory analytical tools for planning. These include expert interviews and group discussions to determine needs in the district [34, 40], social environment analyses and evaluation of social environment-related data for developing measures [29, 44], as well as qualitative interviews, qualitative network analyses, and activating surveys. The importance of planning processes is particularly emphasised in five articles. Larsen et al. [38], for example, develop a tool for the assessment of already existing municipal health-related measures that can particularly be used to the further development of these. Storm et al. [43] present a maturity model for assessing of local HiaP-strategies, planning of measures and monitoring progress (MM-HiaP). In contrast to Kegler et al. [37], who emphasise the importance of planning processes for the success of intersectoral cooperation but do not derive any concrete recommendations for action, Böhme et al. [32] conclude with action guidelines for interdepartmental planning processes. Magnus et al. [39] used 'Search Conferences' as a practical method of access to participation-oriented planning of municipal health promotion and prevention.

The term ‘establishment of structures’ is used to describe measures whose concepts overcome access barriers to services and create new service structures within the municipality or neighbourhood in order to integrate difficult to reach groups. Seven of the fifteen included publications mention this type of structural formation in their research reports [29, 30, 34, 35, 40, 41, 44]. In all seven publications there are also approaches for raising awareness for health-related topics and ongoing offers aiming encounter or exchange. Health days for example can provide a central impulse for networking [29] or peer-organised excursions for the residents of the neighbourhood can be linked to health-related topics [30]. Two publications name active involvement of residents as multipliers as an option for structural formation [30, 34].

### Available evidence on the effectiveness and implementation of community health promotion and prevention

The second research question focuses on the available evidence on the effectiveness and implementation of municipal health promotion and prevention. The inclusion of the term ‘evidence’ in the research strategy was achieved by defining further search terms such as quality, good practice, etc. (see method section). Publications that were designed as research projects or presented scientifically evaluated practical projects were included in the selection. For an overview concerning the evaluation tools applied in in the projects, see Table 3.

**Table 3:** Evaluation tools applied in the included projects

Publications	Evaluation tools
Alisch and Freytag-Leyer [29]	Action research approach to the deduction of events (health days) and strategic alliances, activating survey and qualitative network analysis
Berg et al. [30]	Surveys of several partners, focus groups, documentation forms
Böhme et al. [32]	Explorative research approach with interviews, 2 expert reports, 5 case studies in different cities, document analyses, symposium and business simulation, triangulation method qualitative research approach
Frantz and Heinrichs [33]	Questionnaires regarding strengths and weaknesses, KINDL-R for health-related Quality of Life (non-randomised controlled study)
Große et al. [34]	Mixed methods by means of expert interviews, focus groups, questionnaires for residents etc
Hargreaves et al. [35]	Ethnography, qualitative interviews, group discussions, team surveys, activity tracking
Harris and Sandor [36]	Delphi Study
Kegler et al. [37]	focus groups, semi-standardised interviews with local health care actors and coordinators
Larsen et al. [38]	EUPHID (European Community Health Promotion Indicator Development) model as starting point, expert interviews, focus groups, development of criteria for types of classification
Magnus et al. [39]	Case study, ethnography, written open survey
Reimann et al. [40]	Network surveys, case studies, observations, group discussions
Rütten et al. [41]	Pre, post and follow-up surveys, cognitive tests, qualitative content analyses of planning meetings and documents, checklists, observations
Steenbakkers et al. [42]	Mixed-method approach with online surveys, in-depth interviews, activity tracking
Storm et al. [43]	Developed classification model "Maturity Model for HIAP" (MM-HIAP), document analyses, interviews, online surveys
Trojan et al. [44]	Expert surveys, questionnaires, group discussions, social space analyses, comparative evaluation by means of the framework concept ‘capacity development in the quarter’ („Kapazitätsentwicklung im Quartier “)

The qualitative content analysis of the included publications shows that the concept of evidence is not regularly applied in the development and evaluation of good practice in health promotion. Only five of 15 publications use the term "evidence" directly [33, 35, 39, 41, 42]. With regard to classes of evidence as defined by the Canadian Task Force [46], differences between the analysed publications are apparent. Frantz and Heinrich [33] try to meet the highest standards. Their dissemination study follows a controlled comparative design and thus aims at results in an evidence class II-1. In the final appraisal of their results, they argue that conditions of controllability for their chosen study design were difficult to impossible to achieve. Further, Frantz and Heinrich [33] evaluate the evidence-based selection of competence trainings as critical. Regardless of their evidence base, these trainings apparently were not geared towards the needs of parents and children and should have been adapted accordingly. Evidence alone therefore does not seem to be a guarantee for the acceptance of an intervention by the target group.

They critically discuss the exclusivity of scientific, evidence-based programmes in the context of health promotion. Nevertheless, at the same time, they also reject a solely practice-oriented approach in health promotion. They therefore advocate a middle course by means of a participatory theory–practice partner process. This process could be implemented particularly in the interactive knowledge transfer between the participants. It could further enrich the discussion around varying underlying notions and the lack of quantifiable evidence in the health sciences. Rütten et al. [41] do not speak in favour of a general rejection from evidence-based programmes. They argue that sustainable programme implementation rather should be based on the proven effectiveness of evidence-based programmes in practice. This can be achieved through continuous interactive knowledge exchange and the willingness to adapt programmes to meet practical requirements.

When comparing the previously outlined work of Frantz and Heinrichs [33] and Rütten et al. [41] with the work of Hargreaves et al. [35], Steenbakkers et al. [42] and Magnus et al. [39], two parallels emerge with regard to approaches to the concept of evidence. With their "Healthy Weight Cooperative" Hargreaves et al. [35] also aim at evidence-based programmes for the prevention of overweight. Based on reports on the effectiveness of intervention programmes similar in theme, they developed a new model for evidence-based obesity prevention. Since they do not refer to the term ‘outcome’ in the sense of the effect of measures on the primary target group, but rather use ‘output’ in the sense of the number of measures implemented, no clear position on evidence can be derived from this work. Steenbakkers et al. [42] and Rütten et al. [41] however, take a rather critical view to an evidence classification that is based on clinical studies within the framework of the 'Health in all Policies' (HiaP) approach. Following them, the method of the 'Health Impact Assessment', for example, has not proven to be a useful tool for making decisions and creating measures within the context of the HiaP objective. This was due to the incompatibility of evidence-based measures with the structures found in municipal practice. Taking this into account, Steenbakkers et al. [42] developed a coaching programme for municipal actors. However, due to numerous uncertainties regarding implementation and resulting challenges of the assessment and evaluation, this programme also does not allow for appraisal of its effectiveness.

Against the background of the concept of evidence, Magnus et al. [39] describe the use of search conferences as a planning method. They equate "evidence-based" measures with "participatively developed" interventions. They are not alone in this attribution of the importance of evidence-based interventions, as the included publications show a high level of agreement with participatory approaches. This is accompanied by the assumption that a participatively developed offer could be associated with improved compliance with measures by the target group.

The remaining publications do not directly refer to the concept of evidence. Nevertheless, they also address issues of the effectiveness of community health promotion and prevention. Storm et al. [43] designed a comparative study to develop and test their maturity model of the HiaP strategy at the municipal level. The aim was to obtain first indications of the effectiveness of the framework model. The Delphi study by Harris and Sandor [36], as a primarily qualitative approach, can be assigned to the rather low class of evidence III (expert knowledge). The same applies to the work of Larsen et al. [38], who conceptualised a measure assessment by means of a case study and examined its effectiveness in a Danish municipality. Kegler et al. [37] developed a mixed-method design to identify contextual factors for successful planning of measures. However, the results cannot be assigned to a class of evidence in the classic sense either.

None of the publications indicate attempts to evaluate the effectiveness of practice-based approaches to community health promotion in terms of higher classes of evidence. Trojan et al. [44], for example, developed an evaluation instrument for the district project 'Lenzgesund'. However, only evidence class III can be assigned to this instrument.

All other evaluation reports on district projects or district development approaches are to be classified in the same way. In many cases, the authors nevertheless refer to the effects of the interventions. For example, Reimann et al. [40] developed quality criteria and Böhme et al. [32] presented guidelines for interdepartmental planning in a comparable way. Berg et al. [30] present a detailed extensive evaluation design in their final evaluation of the ‘City district mothers’ project. Within this frame, they address the numerous positive effects of the measures reported by non-professionally trained helpers and mothers in the city district. Große et al., Menkouo [34] and Alisch and Freytag-Leyer [29] also present examples of good practice. In these, an attempt was made to plan measures based on approved qualitative research methods and to measure possible successes with the help of evaluation methods.

In summary, none of the included publications draw conclusions for clear effects on target groups according to a medical definition/approach of the concept of evidence base.

## Discussion

With regard to the theoretical models and approaches of municipal health promotion, the results of the literature and database research show a diverse implementation of strategies and measures. Capacity building, planning and the establishment of structures were identified as applied theoretical models and approaches. Furthermore, the included publications often emphasise the importance of intersectoral networking [33, 41, 42] for improvement of neighbourhood-related health promotion and more environmental justice in cities [32]. The 15 included evaluation studies and research reports were examined regarding the available evidence on the effectiveness and implementation of the strategies of municipal health promotion. In the context of municipal health promotion, the term ‘evidence’ and its underlying definition differ from the definition and usage of the term within the medical research context. Only few of the publications included could meet the standard of evidence classes as established in the classical medical context.

In addition to the theoretical models and approaches identified in the included publications, an overall municipal health and prevention concept can be a basis for a targeted and coordinated overall strategy of municipal policy [26]. This is supported by Böhm [24]. According to this, there are numerous health promotion measures in many municipalities. However, these are not very target-oriented and only partially aligned with the respective needs and requirements in the community. The mix of behavioural and environmental prevention intended by the HiaP-strategy is not always followed in the included publications either. So far, the municipality often serves only as a starting point for interventions, but not as a field of activity for designing preventive measures [24]. With municipal health promotion strategies and concepts, citizens can be reached in a more target-oriented way and double structures in the municipality can be avoided. They can also lead to greater transparency and awareness [24]. Many of the included publications focus on the planning and implementation of municipal health promotion strategies. This indicates research interest in this area. This interest may be based on the idea of efficiency, as health promotion measures have to be planned precisely and aligned to needs in view of tight budgets in municipalities.

In only five of the 15 included publications the term ‘evidence’ is used explicitly. In these publications the definition of the term is not based on the concept of evidence as applied in medical research [33, 35, 39, 41, 42]. In addition to the varying definitions, challenges in the application of appropriate research designs and formats (e.g. the conditions of controllability) can occur, hindering the generation of evidence for effects in higher classes of evidence [33]. Furthermore, the effectiveness of individual measures can probably be demonstrated, but that does not automatically result in sustainable practice implementation and adaptation to local requirements [33, 41, 42].

The varying definitions of the term’evidence’ represent enormous challenges for municipal health promotion: Following the developments in evidence-based medicine in recent years, the demand for evidence-based health promotion has become increasingly apparent. However, medical and therapeutic interventions and health promotion interventions often differ from each other, for example in the interpretation of the concept of health and disease. Health care traditionally focuses on alleviation of undesirable health conditions or specific diseases. Interventions often directly affect physical and psychological processes (e.g. through medication or surgery) and effects become visible immediately [46, 47]. Accordingly, it is not surprising that the understanding of the term ‘outcome’, which is used in the context of municipal health promotion, differs from the classically medical connotation of the term. Hence, it is not used in the sense of a unit for effectiveness, but rather as the number of resulting measures. It is difficult to make statements about the outcome effectiveness in health promotion, because results take time to emerge [35, 46–48].

In the context of health promotion, the concept of health is based on a broader understanding. “Health is the state of complete physical, mental and social well-being" [49] and thus encompasses much more than just absence of disease and infirmity. Accordingly, health promotion and primary prevention interventions attempt to influence the health of individuals in a complex way indirectly through their behaviour and the surrounding conditions, such as internal, interpersonal, and organisational factors as well as the built and natural environment [50]. In this way, municipal health promotion offers the opportunity to reach all population groups at a low-threshold and to promote health equity [30, 34, 49].

Among the differences regarding the understanding of the term ‘evidence’, there is a lack of research designs and formats allowing for the generation of evidences in the municipal health promotion. Study designs such as randomised controlled trials (RCTs) are considered the gold standard in clinical research for demonstrating potential efficacy [51]. None of the included publications describe study designs and methods that could be used to obtain evidence on the effectiveness of interventions. This may be due to the complexity of the municipal living environment and a possible interaction of individual settings within this field. Hence, successful approaches to health promotion and prevention can undoubtedly be implemented in this field, but the application of a controllable study design remains a challenge [51]. Consequently, possible effects of health promotion strategies may not be operationalised nor be quantifiable. The complexity of the factors that affect the community's inhabitants stand in contrast to the laboratory conditions that are possible in some areas of medical health research. It is almost impossible to isolate the effects of individual measures in the field of municipal health promotion. Even in the case of individual behavioural interventions, the proof of possible effects is already complex, since these interventions usually have indirect effects that are not achieved directly [51]. Such proof becomes more difficult with increasing complexity as it occurs in overall strategies that are intended to have behavioural and environmental effects. Furthermore, an effect often solely appears in the long term [35]. This makes it difficult to clearly attribute the cause. It is possible that target groups have already been exposed to a variety of other social and environmental factors in the meantime, which can change the health-promoting effects of the implemented measures. Due to this complexity and dynamics, it is not possible to assume simple linear causal chains between the health-promoting interventions and the effects on individual health [35, 51].

The methodology used to generate the present results has some limitations. Thus, publications representing guidelines for action for the implementation of municipal health promotion and further innovative programmes were also reviewed [52–54]. However, these could not be included due to the inclusion and exclusion criteria defined in advance. During the research process, numerous other references to municipal projects were also registered, some of which outlined innovative intervention approaches, but did not take into account any evidence or similar criteria (evaluation, quality assurance, etc.) in their presentation of municipal measures and strategies. Thus, the final selection of results is based on the composition of the search operators and their close interaction with evidence and related terms.

## Conclusions

The examined publications reveal a desire to examine measures and interventions of municipal health promotion in terms of their quality, mechanism of action and effectiveness. Therefore, recognised evaluation methods are used, context and success factors are identified, and elaborate evaluation designs and guidelines are conceived, some of which are based on the gold standard of medical research. However, it is also apparent that these research designs can be transferred to municipal health promotion only to a limited extent. Although the present scoping review cannot close the gap of knowledge regarding theoretical models and approaches and the evidence of municipal health promotion and prevention, it allows for recommendations for action that enable evidence-oriented planning, implementation and evaluation of strategies of municipal health promotion [26].

The following eight recommendations for action can be derived from the presented results. To promote health equity, it is important to encourage and facilitate planning processes in urban and neighbourhood development. A second recommendation is reinforcement and support of strategic alliances in terms of development and maintenance thereof in order to build networks that can diminish health inequalities. Furthermore, it is important to ensure the citizens’ participation in the development and implementation of measures. In order to reach everybody, an important strategy is the development of low-threshold approaches to health promotion in the municipality and the systematic integration of local partners. The fifth recommendation for action is anchoring measures of health promotion and prevention in existing municipal structures so that an intersectoral establishment of measures in the sense of HiaP can succeed. Another recommendation is to strengthen theory development and research for the continued development of health promotion. Therefore, it is advisable to involve stakeholders from academia. Municipal health promotion should be aligned with sustainability. In addition, evidence-based health promotion needs to be programmatically realigned to address the complexity of multilevel strategies and to generate evidence.

## Supplementary Information

**Additional file 1.**

## Data Availability

Data sharing is not applicable to this article as no datasets were generated or analysed during the current study.

## Abbreviations

BZgA: Federal Centre for Health Education (German: Bundeszentrale fuer gesundheitliche Aufklaerung)
EUPHID: European Community Health Promotion Indicator Development
HiaP: Health in all Policies
MM-HiaP: Maturity Model for Health in all Policies
PRISMA: Preferred Reporting Items for Systematic reviews and Meta-Analyses
RCT: Randomised Controlled Trial
WHO: World Health Organisation

## Footnotes

1 These databases were selected because we believe they provide a broad overview over relevant publications and projects (esp. the German-language database 'Kooperation für nachhaltige Prävention und Präventionsforschung' provides information especially about grey literature and projects in the field of health promotion research).

2 Form of presentation following Moher et al. (23). Own table according to Quilling and Kruse (22).
