# Supplementary figures and images for: State of evidence on municipal strategies for health promotion and prevention: a literature and database research (Scoping Review)
Source: BMC Public Health. 2022 Feb 14;22:301. doi: 10.1186/s12889-022-12607-0 (PMC8842970; doi:10.1186/s12889-022-12607-0)

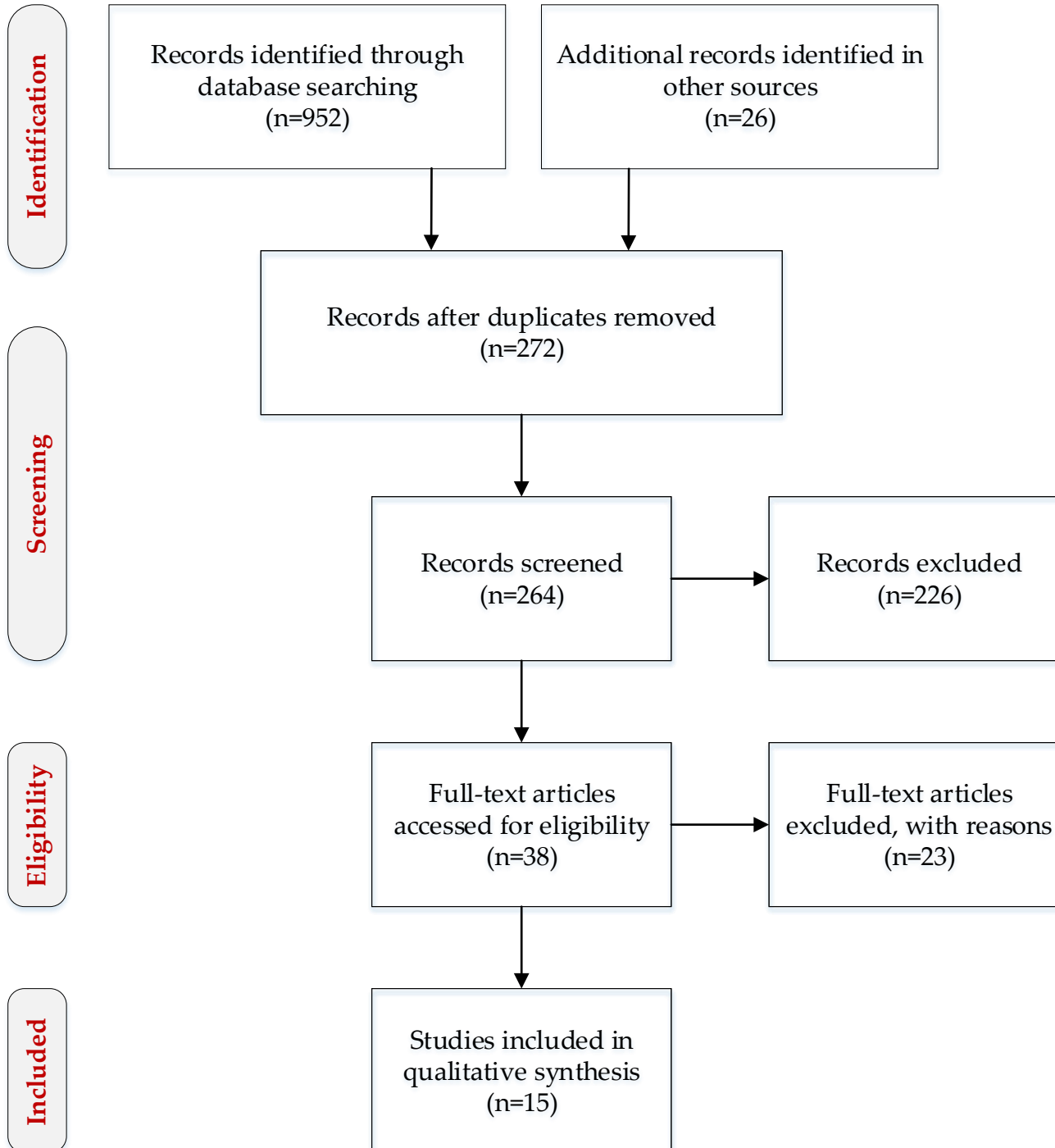

Supplement: Supplementary file 1 — Additional file 1. [file 12889_2022_12607_MOESM1_ESM.pdf]
